# Morphological, Chemical, and Physical–Mechanical Properties of a Clumping Bamboo (*Thyrsostachys oliveri*) for Construction Applications

**DOI:** 10.3390/polym14173681

**Published:** 2022-09-05

**Authors:** Zhenhua Zhang, Fei Rao, Yujun Wang

**Affiliations:** 1 College of Art and Design, Zhejiang Sci-Tech University, Hangzhou 310018, China; 2 Basic Forestry and Proteomics Center (BFPC), College of Forestry, Fujian Agriculture and Forestry University, Fuzhou 350002, China

**Keywords:** *Thyrsostachys oliveri*, culm morphology, chemical component, physical–mechanical properties

## Abstract

In view of the long-term utilization history as a building and furniture making material in southeast Asian countries, *Thyrsostachys oliveri* is considered to have great utilization potential. However, little is known about the quantitative morphological characteristics and comprehensive material properties of its culm. In this study, we systematically investigated the morphological characteristics, the chemical components, and the physical–mechanical properties of the three-year-old culm of *T. oliveri.* The morphological analysis result showed that the internode length, the diameter of internodes and the wall thickness changed with the culm height. The volume of the culm wall of a single internode increased before the 10th internode, and then it decreased to a significant level at the 20th internode. The basic chemical compositions (cellulose, hemicellulose, lignin and silicon content) of the culm wall were 346.19 mg/g, 95.32 mg/g, 33.17%, and 3.39 mg/g, respectively. These component contents were relatively stable in the bottom and middle part of the culm, but changed significantly in the upper part of the culm. The moisture content and the base density of the culm wall were 73.01% and 0.64 g/cm^3^, respectively. The culm wall shrinkage rate in the radial, tangential direction as well as the volumetric shrinkage reached the minimum value in the middle part of the culm. The average compressive strength, modulus of rupture and modulus of elasticity of the culm wall were 67.03 MPa, 143.74 MPa, and 7.99 GPa, respectively. These results provide valuable reference data for more rational use of this bamboo resources.

## 1. Introduction

As a natural organic polymeric material, bamboo has more than 10,000 applications [1,2]. Under the backdrop of the shortage in wood resources today, bamboo has attracted unprecedented attention because of its unique advantages, including its short growth cycle and sustainable harvesting, etc. [3]. The demand for bamboo raw material also has been increasing steadily in recent years [4]. Taking China’s bamboo market as an example, which has the largest bamboo industry in the world, bamboo export and import volume has been increasing since 2015, with only a slight decline in recent years because of the COVID-19 pandemic (https://www.huaon.com/channel/trend/703111.html, accessed on 11 July 2022). Since the demand for bamboo materials at a global scale will continue to increase, we will need to consider the development of bamboo resources to improve its utilization and meet this growing demand.

Bamboo is distributed widely over tropical, subtropical, and mild temperate zones, and has rich species diversity. According to the updated World Checklist of Bamboos, which revised the data published in 2016 in the World Checklist of Bamboos and Rattans [5], 1700 bamboo species belonging to 130 genera have been recorded worldwide [6]. Only a few bamboo species, however, have been wildly planted and effectively utilized. For example, the raw material of China’s bamboo board processing market depends almost completely on Moso bamboo (*Phyllostachys edulis*) [7]. Many high-quality bamboo resources have not yet been effectively developed or fully utilized. Specifically, some clumped bamboo species, which are widely distributed in southeast Asia and south Asia, are underutilized. Unlike scattered bamboo, clumped bamboo has no developed rhizome system; as a result, it will not expand its growth territory at a large scale, which makes it more convenient for management [8]. Therefore, this type of bamboo has distinct development value.

*Thyrsostachysoliveri* is a moderately large clumping bamboo with tall and straight culms. Its culm also has characteristics of high branches with dense and small leaves, which make the appearance of the whole bamboo cluster slender and elegant. The *T. oliveri* was only regarded as a tropical ornamental bamboo in China, but actually, it has been widely used as a local source of material for construction in some southeast Asian regions for a long time [9]. The *T. oliveri* is naturally distributed wildly in southeast Asian countries, such as Myanmar and Thailand, as well as in the Yunnan province of China [10]. At present, it has been introduced and cultivated in other southern provinces of China, such as Fujian, Taiwan, and Guangdong Province. Unfortunately, little is known about the culm morphological characteristics and basic physical and chemical properties of this bamboo species, which hinders the development of its material value to a great extent. In this study, a series of morphological analyses were carried out on this bamboo species for determining its morphological characteristics. Furthermore, in order to further enhance its utilization value, the chemical proponents and basic physical–mechanical properties of its culm have also been tested and analyzed; these results will be necessary to evaluate its most suitable use and will assist in the utilization and processing of this bamboo species.

## 2. Materials and Methods

### 2.1. Sampling Location and Environment

Bamboo samples were obtained from the Bamboo Garden of Huaan (25°00′36″ N, 117°32′27″ E), which is located in the Fujian province of China. The sampling site is 150 m above sea level, with an annual average temperature of 17.5 °C and an average annual precipitation of 1447.9 mm. It is located in the climate transition zone between the middle subtropical and south subtropical zones. 

### 2.2. Determination of Culm Morphological Characteristics

To determine the morphological characteristics, we selected 30 three-year-old bamboo culms from 10 bamboo clumps and measured their diameter at breast height (DBH). We calculated the average DBH value based on the previous data and randomly selected five healthy and complete bamboo three-year-old culms as the standard samples from those 30 culms. Then, we measured the culm height (CH), culm height under the branches (CHB), and ground diameter (GD) of the standard bamboo culms after they were harvested from the culm base. Subsequently, we measured the length, diameter, and wall thickness of each internode.

### 2.3. Determination of Chemical Composition

**Figure 1:**
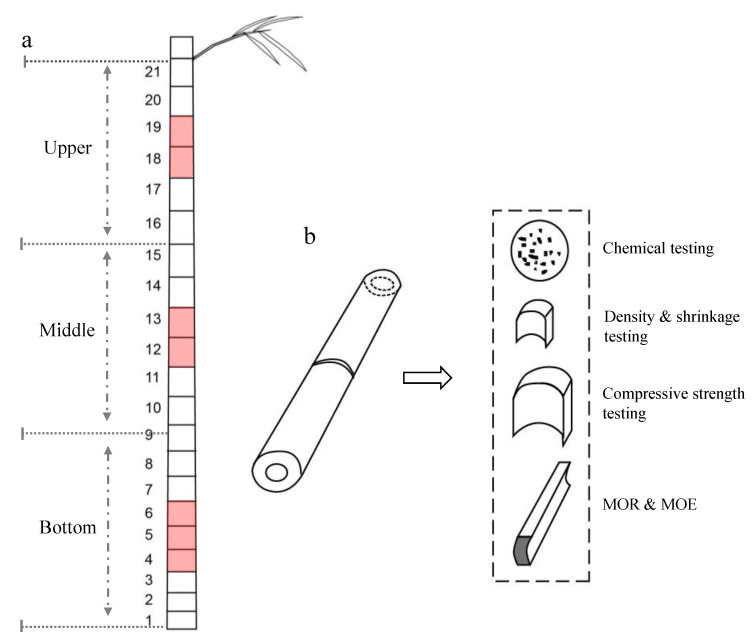
Schematic diagram of sample selection and making. (**a**) The location of the internode where the testing sample was cut. The internodes marked with pink are the parts which represent the bottom (the 4th–6th internodes), middle (the 12th–13th internodes), and upper (the 18th–19th internodes) parts of culm, respectively. (**b**) Sample making for chemical component and physical–mechanical testing.

We selected the culms below the first branch of the standard bamboo. We divided them into three equal parts (the upper, middle, and bottom parts) according to the total length. Two to three internodes located in the middle of each part were further cut into a corresponding shape and size as the samples for subsequent chemical and physical–mechanical experiments, respectively (Figure 1a).

The samples for chemical composition determination were dried and ground into powder (Figure 1b). The samples from each culm position had three biological replicates. The determination of the cellulose content was measured using a referenced method [11,12]. The sample was first weighed accurately at 0.1000 g, and then it was heated and hydrolyzed under acidic conditions. The cellulose content was determined and calculated according to anthrone–sulfuric acid colorimetry. The content of hemicellulose was determined by the dinitrosalicylic acid (DNS) reducing sugar method, according to the detailed detection steps referenced by Chen [13]. For lignin content detection, according to the method described in Pochinok [14], we used 72% concentrated sulfuric acid to first remove the cellulose and then indirectly calculate the lignin content by the oxidation reaction. The silicon concentrations in the samples were determined by the colorimetric molybdenum blue method, which has been described in detail by Hua et al. [15]. 

### 2.4. Examination of Culm Physical–Mechanical Properties 

The size of samples (kept fresh and wet) for the density and shrinkage rate testing was 10 mm (longitudinal) × 10 mm (tangential) × *t* mm (radial, i.e., wall thickness). For the compressive strength (CS) testing, the size of the sample was 20 mm (longitudinal) × 20 mm (tangential) × *t* mm. For modulus of rupture (MOR) and modulus of elasticity (MOE) testing, the size was 10 mm (longitudinal) × 160 mm (tangential) × *t* mm (Figure 1b). We used the remaining standard samples for moisture content determination. The physical properties of the bamboo material, including moisture content, density, and dry shrinkage, were determined using the testing methods for physical and mechanical properties of bamboo (GB/T15780-1995 procedures) [16]. We included at least 10 sample replicates in each experimental group.

The mechanical properties of the *T. oliveri* were measured by a universal testing machine (5969R6302, INSTRON, Norfolk County, MA, USA) with 50 kN load cell. The compressive strength (CS), modulus of rupture (MOR), and modulus of elasticity (MOE) of these were tested according to the standard GB/T15780-1995. The number of test specimens of CS was 35.

The Compressive Strength (CS) was calculated using Equation (1), as follows:

(1) $$\mathrm{CS}\left(\mathrm{MPa}\right)=\frac{P_{max}}{bt}$$

where *P_max_* is the maximum failure load of the sample, *b* is the width of the sample, and *t* is the thickness of the sample (bamboo wall thickness).

The tree-point bending test was conducted to determine the MOR and MOE of bamboo specimens. The MOR and MOE were calculated using Equations (2) and (3), respectively.

(2) $$\mathrm{MOR}\left(\mathrm{MPa}\right)=\frac{3P_{max}L}{2bh^{2}}$$

(3) $$\mathrm{MOE}\left(\mathrm{GPa}\right)=\frac{PL^{3}}{4bh^{3}f}$$

where *P_max_* is the maximum failure load of the sample, *L* is the span between two supports, *b* is the width of the sample (Bamboo wall thickness), *h* is the height of the sample, *P* is the difference between the upper and lower load, and *f* is the deformation increases in the middle of the sample.

## 3. Results

### 3.1. Morphological Characteristics

**Figure 2:**
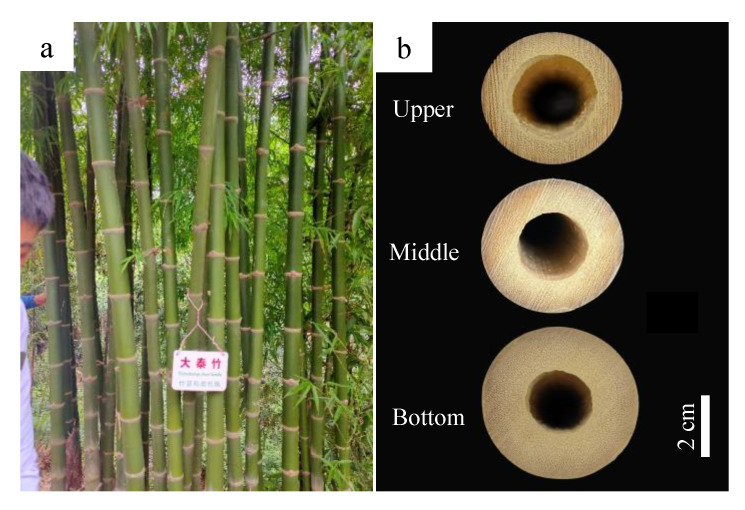
The culm morphological characteristics of the *T. oliveri*. (**a**) Appearance of culms below branches; (**b**) the cross section of the culm.

The culms of *T. oliveri* are straight with high branches and thick culm walls (Figure 2a, b). Morphological statistics results show that its average CHB reached 6.87 m, accounting for half of the average culm height (CH, 12.9 m) (Table 1). In the field investigation, we found that the CHB value varied significantly among different bamboo clumps. The CHB of those bamboo clumps with low culm density (after manual pruning and felling) often was much lower than that of those with high culm density. The average diameter at breast height (DBH) and the ground diameter (GD) of the culm reached 4.88 cm and 5.77 cm, respectively. The average internode length (AIL) and the average wall thickness (AWT) of the culm under the first branch were 26.78 cm and 11.51 mm, respectively (Table 1). 

### 3.2. Variation Pattern of Bamboo Culm Morphology

**Figure 3:**
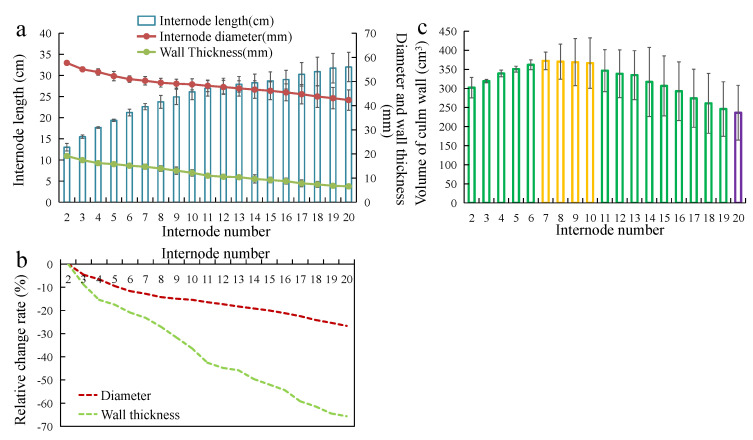
Variation pattern of bamboo culm morphology in the *T. oliveri.* (**a**) The length, diameter, and wall thickness of the internodes from the base 1st to 20th. (**b**) Relative change rate of the internode diameter and the wall thickness with the internode number. (**c**) Volume of culm wall of internodes from the base 1st to 20th. The green, yellow, and purple bars indicate significant differences among the three (*p* < 0.05).

The culms below the branches usually are the main part used for processing bamboo material. We analyzed the morphological variation trend of this part of *T. oliveri* (from the base of the 2nd internode to the 20th internode) (Figure 3a, b). The results showed that the internode length increased with culm height before the 20th internode, but the internode diameter and wall thickness followed the opposite trend (Figure 3a). Note that, compared with the internode diameter, the downward trend (the relative changes rate) in wall thickness with the culm height (the increase in the node number) was more intense in *T. oliveri* (Figure 3b). Since these three morphological indices are closely related to the volume of culm wall, therefore, we further analyzed the change trend of the culm wall volume of the internode, with the internode number increasing in *T. oliveri*. The results showed that the volume of the culm wall increased first and then decreased as the internode number increased, and the largest culm wall volume appeared in the 7th to 10th internode, which were significantly larger than those of the other internodes (Figure 3c).

### 3.3. Chemical Composition of Bamboo Culm in T. oliveri

In *T. oliveri*, the average content of cellulose, hemicellulose, and lignin in the culm under the branch was 346.19 (mg/g), 95.32 (mg/g), and 33.17 (%), respectively (Table 2). The content of cellulose in the upper part of bamboo culm was significantly higher than that in the middle and lower part. This followed a similar trend to the changes in lignin but followed the opposite trend to the changes in hemicellulose content (Table 2). In addition, we detected the silicon content of bamboo culm, which was another important composition that can affect bamboo proprieties. The results showed that the average value of silicon content in the culms under the branch was 3.39 (mg/g). The content of silicon in the upper part of bamboo culm was greater than in the middle and lower parts (Table 2).

### 3.4. Basic Physical Properties of Bamboo Culm in T. oliveri

**Figure 4:**
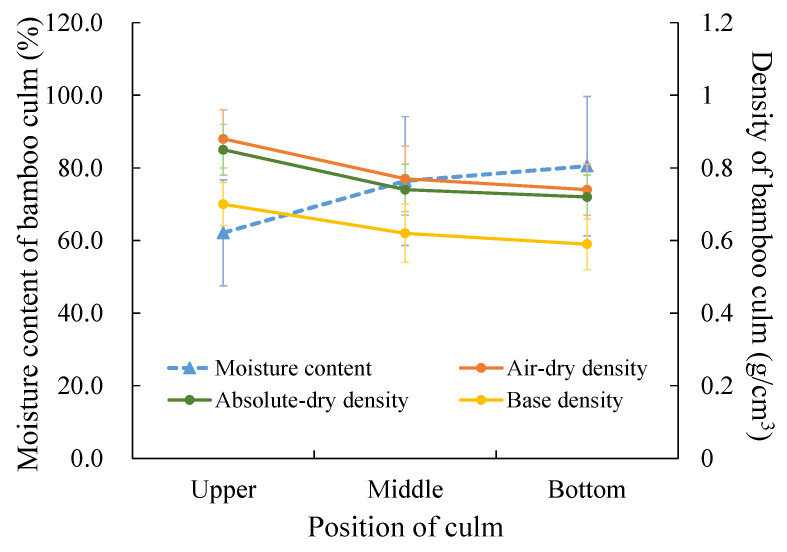
The moisture content and densities of the culm wall in different culm position.

Moisture content, densities, and dry shrinkage are the three basic physical properties of engineering material, and are also the main factors affecting the dimensional stability and mechanical properties of bamboo materials. The moisture content of the part under the branch in *T. oliveri* averaged 73.01% and decreased from the upper side to the bottom of the culm (Table 3; Figure 4). The culm wall densities in the air-dry and the absolutely-dry conditions as well as the base density were 0.80 g/cm^3^, 0.77 g/cm^3^, and 0.64 g/cm^3^, respectively, and they have a converse change trend to the moisture content (Table 3; Figure 4). 

**Figure 5:**
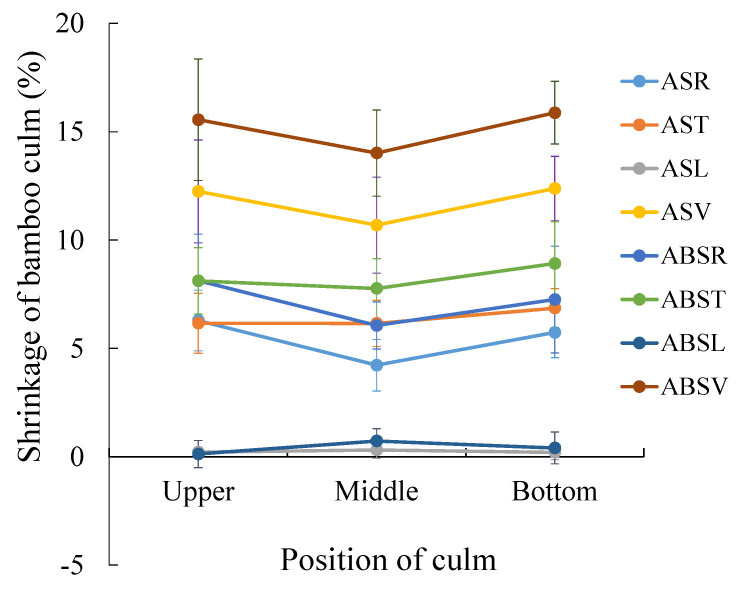
The shrinkage rate of the culm wall in different culm position. ASR, air-drying shrinkage in radial direction; AST, air-drying shrinkage in tangential direction; ASL, air-drying shrinkage in longitudinal direction; ASV, air-drying shrinkage of volume; ABSR, absolute-drying shrinkage in radial direction; ABST, absolute-drying shrinkage in tangential direction; ABSL, absolute-drying shrinkage in longitudinal direction; ABSV, absolute-drying shrinkage in volume.

The average shrinkage rates of the culm wall volume at air-drying and absolute-drying condition were 11.77% and 15.15%, respectively, and the minimum values appeared in the middle of the culm in *T. oliveri* (Table 4; Figure 5). In different directions of the culm wall, the tangential shrinkage rate (6.39% in air-drying condition and 8.27% in absolute-drying condition) had the largest values, whereas the smallest shrinkage rate appeared in the longitudinal direction. Similarly, the dry shrinkage rate in the radial and tangential in the middle of the culm was less than that of the upper and lower parts in the *T. oliveri*. This variation trend reached a significant level in the radial direction of the culm (Table 4; Figure 5).

### 3.5. Mechanical Properties of Bamboo Culm of T. oliveri

The average compressive strength, MOR, and MOE of the culm wall in the *T. oliveri* were 67.03 ± 9.26 MPa, 143.74 ± 19.16 MPa, and 7.99 ± 2.60 GPa, respectively (Table 5). In the middle of the culm, the compressive strength of the culm wall was the smallest and was significantly smaller than that of the upper part of the culm. The MOR followed an increasing trend with culm height but did not reach a significant level. The MOE value of the culm wall at the base of the culm was the smallest (5.55 ± 1.19 GPa) and was significantly lower than that of the middle and upper part of the culm (Table 5).

## 4. Discussion

The morphological characteristics and size of bamboo culm were important reference indexes to evaluate their utilization and processing methods. For *T. oliveri*, we identified two morphological characteristics worthy of attention. One was its thick culm wall, which could reach more than 20 mm at the base of the culm. Its wall thickness was much greater than the average wall thickness of Moso bamboo, which is rare in bamboo spices with the same culm diameter. The thickness of the culm wall, however, greatly decreased with an increase in the culm height in *T. oliveri* (Figure 3b), which likely significantly reduced the volume of the material in the upper part of the culm and affected the material processing. Therefore, we analyzed the change trend in the culm wall volume of the internode at different culm heights. The result was consistent with our expectations that the culm wall volume decreased significantly from the 21st internode. Interestingly, for *T. oliveri*, the 21st internode was the position at which the branches started to grow (about 6.87 m from the culm base), although most of the culm with branches would be removed during harvesting to improve efficiency. Another morphological characteristic was that the culm of *T. oliveri* was straight and uniform. The culm diameter changed slightly with culm height in the culm under the first branch, which was favorable for processing.

Cellulose, hemicellulose, and lignin are the three main components of the cell wall, the substantial bearing structure of woody raw materials. In bamboo wood, they account for more than 90% of the total mass [17]. From the perspective of composite materials, the cell wall can be regarded as a polymer, with lignin and hemicellulose as the matrix and cellulose as the reinforcing phase [18]. Therefore, their content and proportion will directly affect the mechanical properties of cell walls and then affect the physical–mechanical properties of bamboo materials. According to reports, the cellulose content in woody bamboo is very close to that of softwood (40–52%), which is in the range of 40–50% [19]. Raw materials with cellulose content in this range are more suitable for papermaking [20]. The average cellulose and the hemicellulose content of *T. oliveri* culm was 346.19 mg/g and 95.32 mg/g, respectively (Table 2), which accounted for only about 35% of the total biomass. This content value was closer to that of some hardwoods with low cellulose content (the cellulose content of hardwoods ranged from 38 to 56%) [19]. The average lignin content of the *T. oliveri* culm reached more than 33% (Table 3), which was much higher than that of common bamboo (20–26%) and was close to the content range of North American softwoods (24–37%) [17,21]. In the cell wall, hemicellulose cross-links the lignin and cellulose to form a mesh-like structure, endowing wood with strong structural rigidity [22]. Moreover, the high hydrophobicity of lignin reduces the water absorption of woody material to a certain extent, thus enhancing its dimensional stability [23]. The high lignin content of the *T. oliveri* suggested that it may have strong dimensional stability. In addition, bamboo is a typical silicon super enriched plant [24,25]. Silicon in bamboo can further enhance the structural rigidity of its cell wall and effectively resist diseases and pests [26]. The average silicon content of the *T. oliveri* culm was 3.39 mg/g (Table 2), in the middle level of the range of common bamboo species (1.77 mg/g to 5.18 mg/g) [27,28]. These chemical components were significantly larger in the upper part than in the middle and lower parts of the culm of *T. oliveri*, except for hemicellulose (Table 2). This result may be attributed to a significant reduction in bamboo wall thickness in the upper part of the culm.

The physical–mechanical properties were the key indicators to evaluate the applicability of bamboo materials, especially in the field of construction applications. According to a report covering 141 species of bamboo (excluding the object of this study), the moisture content of fresh bamboo varies greatly among different species (usually 80–100%) [29]. The average moisture content of the 3-year-old culm of the *T. oliveri* was 73.01% (Table 3), which is at the low or middle level when compared with the more than 100 species above. Its relatively lower moisture content may be related to the high lignin content in its culm wall, and, at the same time, this may also make *T. oliveri* culm have a higher basic density (0.64 g/cm^3^) (Table 3), which is higher than that of Moso bamboo (0.61 g/cm^3^) of the same culm age [29]. As a candidate material for construction, *T. oliveri* culms have no obvious advantage in size; thus, it may be more suitable as a processing unit. Therefore, we compared the physical–mechanical properties of the culm wall in different culm positions, considering the importance of material homogeneity. The radial and tangential shrinkage and volume shrinkage of the middle part of the culm were less than those of the upper and base parts in the *T. oliveri*. This variant pattern was different from Moso bamboo, *Neosinocalamus affinis*, and other clump bamboos [8,30,31], but did not reach a significant level, which indicated a good homogeneity. Moreover, the culms of the *T. oliveri* have strong compressive properties. Its compressive strength (67.03 ± 9.26 MPa) is better than that of the culm wall of Moso bamboo at same culm age (59.54–65.97 MPa) [32,33]. In addition, the culms of the *T. oliveri* also have excellent bending resistance. The modulus of rapture of its culm wall reaches 143.74 ± 19.16 MPa, which is significantly better than the culm wall of Moso bamboo at the same culm age (109.13 MPa) [32]. The excellent mechanical properties of the culm of the *T. oliveri* may be related to the high content of lignin in its culm wall (Table 2).

In summary, our research comprehensively analyzed the comprehensive characteristics of the culm material of *T. oliveri* according to three aspects: morphology, chemistry, and physical-mechanics. The most effective utilization part of *T. oliveri* is the culm section from the 1st to 20th internodes. Lignin accounts for a high proportion of the chemical composition of the culm wall, which may give its bamboo culm excellent physical and mechanical properties. These above results provide valuable reference data for more rational use of this bamboo resource. 

## Figures and Tables

**Table 1:** Basic morphological parameters of culm of the *T. oliveri*.

	CH (m)	CHB (m)	DBH (cm)	GD (cm)	AIL (cm)	AWT (mm)
Mean-value	12.90	6.87	4.88	5.77	26.78	11.51
STDEV	1.10	1.44	0.64	0.04	1.77	3.96

CH, Culm Height; CHB, culm height under the branch; DBH, diameter at breast height; GD, ground diameter; AIL, average internode length; AWT, average wall thickness.

**Table 2:** Chemical composition of *T. oliveri*.

Position	Cellulose (mg/g)	Hemicellulose (mg/g)	Lignin (%)	Silicon Content (mg/g)
Upper	398.55 b ± 4.91	83.50 a ± 2.92	35.87 b ± 1.45	4.72 b ± 0.45
Middle	319.49 a ± 2.10	101.29 b ± 3.23	33.19 a ± 0.93	2.19 a ± 1.02
Bottom	320.53 a ± 5.03	101.17 b ± 6.28	30.44 a ± 0.69	3.39 ab ± 0.75
Means	346.19 ± 3.45	95.32 ± 9.65	33.17 ± 2.53	3.39 ± 1.84

Values with the same letter(a,b,ab) in the same column are not significantly different at *p* < 0.05.

**Table 3:** The mean values of moisture content and densities of the culm wall.

	Moisture Content (%)	Air-Dry Density (g/cm^3^)	Absolute-Dry Density (g/cm^3^)	Base Density (g/cm^3^)
Means	73.01	0.80	0.77	0.64
STDEV	17.15	0.10	0.09	0.08

**Table 4:** The mean values of shrinkage of the culm wall in different drying conditions.

Day Condition	Radial Shrinkage (%)	Tangential Shrinkage (%)	Longitudinal Shrinkage (%)	Volumetric Shrinkage (%)
Air-dry	5.42 ± 1.49	6.39 ± 1.14	0.25 ± 0.66	11.77 ± 2.13
Absolute-dry	7.15 ± 1.72	8.27 ± 1.35	0.42 ± 0.68	15.15 ± 2.31

**Note:** Value with the same letter in the same column are not significantly different at *p* < 0.05.

**Table 5:** Mechanical properties of the culm wall at different height in *T. oliveri*.

Position	Compressive Strength (MPa)	MOR (MPa)	MOE (GPa)
Upper	69.75 b ± 3.77	148.04 a ± 16.16	8.28 b ± 2.51
Middle	61.45 a ± 11.20	146.71 a ± 20.58	9.04 b ± 2.56
Bottom	68.72 ab ± 10.33	135.69 a ± 20.17	5.55 a ± 1.19
Means	67.03 ± 9.26	143.74 ± 19.16	7.99 ± 2.60

MOR, modulus of rapture; MOE, modulus of elasticity. Values with the same letter (a,b,ab) in the same column are not significantly different at *p* < 0.05.

## Data Availability

Not applicable.
